# Experiment Comparative Analysis of Feed Rate with Velocity Control in Cutting Mono Crystalline Silicon Using a Diamond Wire Saw

**DOI:** 10.3390/mi15040473

**Published:** 2024-03-29

**Authors:** Jiabin Wang, Shujuan Li, Lie Liang

**Affiliations:** School of Mechanical and Precision Instrument Engineering, Xi’an University of Technology, Xi’an 710048, China; wang_ticktack@outlook.com (J.W.);

**Keywords:** silicon, modeling, normal cutting force control, wire saw velocity, part feed rate

## Abstract

Fixed-diamond abrasive wire saw cutting is one of the most common methods for cutting hard and brittle materials. This process has unique advantages including a narrow kerf and the ability to use a relatively small cutting force. In the cutting process, controlling the main process parameters can improve the processing efficiency, obtaining a better processing surface roughness. This work designs the PI controller (Proportional–Integral controller) based on the reciprocating wire saw cutting process. The control objects are the workpiece feed rate and wire saw velocity, and the control objective is the normal cutting force. For the control trials, several reference values of various normal cutting forces were chosen. The effects of feed rate and saw velocity on the cutting surface finish and cutting time were investigated in this work using wire saw cutting analysis on a square monocrystalline silicon specimen. The results of this study showed that under a constant applied force of 2.5 N, the optimal feed rate of the diamond wire through the specimen could reduce cutting time by 42% while achieving a 60% improvement in the measured surface finish. Likewise, optimal control of the wire saw velocity could reduce cycle time by 18% with a 45% improvement in the surface finish. Consequently, the feed speed control is more effective than the wire saw velocity.

## 1. Introduction

Materials such as sapphire, monocrystalline silicon, polycrystalline silicon, and rare-earth permanent magnet materials are widely used in many industries. Invented in 1990, the wire saw is commonly used to cut brittle materials, and very thin and soft wire can cut hard and brittle materials such as optical glass, ceramics, Si, SiC, and even wood. Wire saw cutting with consolidated diamond abrasive grains is the primarily used processing method [1,2]. Cutting a material into wafers is the first process of silicon ingot processing, and 50% of the cutting workload in the cutting process includes cutting, grinding, and polishing [3,4]. Yufei Gao [5] conducted the slicing experiments of single-crystal silicon using a reciprocating electroplated diamond wire saw. Study results show that a higher wire speed and lower ingot feed speed can produce lower wafer SR (Surface Roughness) and SSD (Subsurface Damage); the lower warp of the wafer needs a lower wire speed and ingot feed speed; and low-wafer TTV (Total Thickness Variation) can be obtained by an appropriate matching relationship between wire speed and ingot feed speed. Liu et al. [6] studied the effects of the abrasive density and sawing process parameters on the sawing characteristics. The results showed that the cutting ability and sawing efficiency of the diamond wire saw increased with the increase in the surface abrasive density. Hwang et al. [7] investigated the abrasive wear patterns and the tensile properties of steel saw wires (120 μm in diameter) in order to develop highly reliable saw wires for the application to silicon (Si) ingot slicing.

Bidiville et al. [8] found that the strongest wafers were obtained by using a low abrasive volume fraction in the slurry, a low wire tension, and a slow feed rate. Huang et al. [9] explored the effect of wire vibrations on the materials loss and observed that saw kerf loss and wafer profile error loss were the main forms of the material removal during the slicing process. Wire vibrations have a significant influence on the sliced wafer morphology. Costa et al. [10] studied the influence of diamond wire sawing on the surface integrity of monocrystalline silicon, and the results showed that the surface presented deeper and wider craters with a higher feed rate because of the deeper penetration of the diamond grains. Upon increasing the wire cutting velocity, there were more regions formed in the ductile mode. Liedke et al. [11] studied an instrumented single-wire saw and presented an analytical model for the macroscopic mechanical conditions in the wire sawing process based on experimental results.

Li et al. [12] developed cutting force control for SiC monocrystals using the part feed rate as the control signal. The experimental results demonstrate that the wire saw machining process with adaptive force control can improve the cutting productivity and significantly decrease wafer surface roughness as compared to the cutting process with a constant part feed rate. Additionally, Li et al. [13,14] proposed an analytical force model based on analyzing the forces generated from the chip deformation and friction of a single abrasive; this model was extended to a wire saw cutting force model and was used to explain the relationship between the cutting force and the part feed rate, wire velocity, and contact length between the wire and part. The cutting force changed during the operation due to factors such as changes in the part–wire contact length and the wire direction, variations in the process parameters, and wire wear. The changing cutting force could lead to wire saw breakage, wafer collapse, and increased wafer surface roughness. Wang et al. [15] conducted an experiment for wire saw processing of single-crystal silicon wafers and demonstrated that the novel concept of regulating process forces in wire saw machining by adjusting the wire velocity can be used to optimize the cutting process of single-crystal silicon, making the process more productive while decreasing the part roughness.

Based on the previous research, the workpiece feed rate and the wire saw cutting velocity are the two main process parameters for diamond wire saw processing. This paper analyzes and compares the different effects of the feed rate and wire saw velocity control in the wire saw cutting process. A model was constructed using experimental results, and a proportional–integral (PI) controller was designed with a stable normal cutting force as the control objective. The cutting productivity, wafer topography, and surface roughness are investigated when wire saw machining silicon monocrystal with and without PI normal force control.

## 2. The Experimental Setup and Cutting Force Controller Design

### 2.1. Experiment Setup

For the experimental investigation of the effects of the feed rate and wire saw velocity, we built a hardware system platform and completed the construction of the experimental platform. The basic parameters of the wire saw machine shows in Table 1.

**Figure 1:**
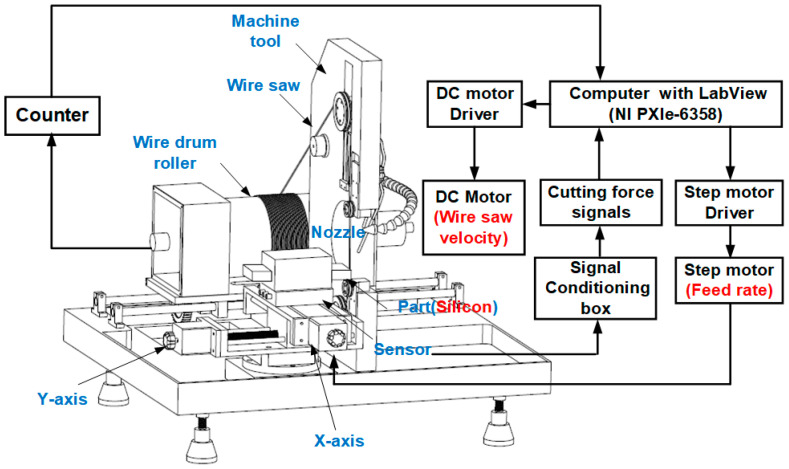
Control experiment system schematic.

Figure 1 is a schematic diagram of the control experiment of the wire saw cutting machine. The part is made of mono-crystal silicon with a size of 220 × 20 × 26 mm, the density is 2.33 g/cm^3^, and the Mohs hardness of polysilicon is about 6.5. The contact length between the wire saw and the part is 26 mm. The wire saw base was made from a high-quality stainless steel and was nickel-plated with a JR2-type diamond abrasive with an average abrasive grain size of 30–50 μm. The wire had a length of 106 m, an average diameter of 0.24 mm, the velocity range was 0–4 m/s, and the feed rate range was 0–3 mm/min. The adjustment wheel could regulate the wire tension in the range of 0–0.6 MPa.

A Gamma SI-32-2.5-type six-component dynamometer from the ATI Company was used to measure the normal force. It has a range of 32 N and a resolution of 1.25 × 10^−2^ N. As Figure 1 shows, the workpiece is fixed with the dynamometer through a special fixture. The dynamometer provides multichannel analog voltage output, the signal is obtained through board card acquisition, and the data of different directions of force in the processing process are obtained after processing. A National Instruments M-series PXIe-6358 multifunction data acquisition system is used to collect and process the data, and the sample frequency is 10 Hz.

### 2.2. Normal Force Model and Controller Design

#### 2.2.1. Normal Force Static Model

**Figure 2:**
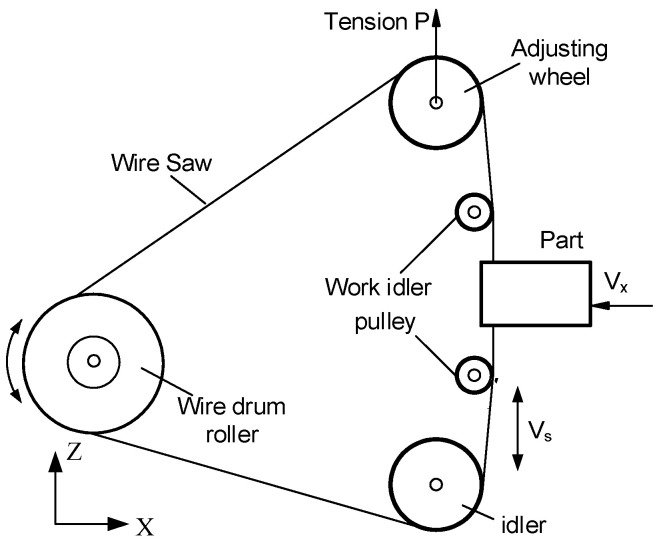
The schematic diagram of wire saw cutting.

The main factors affecting the cutting force in the wire saw machining process are the part feed rate, wire saw velocity, the contact length of part and wire, and the wire tension. Figure 2 shows the schematic of the wire saw for cutting single-crystal silicon. The system consists of a wire wound around the pulley, two pulleys, and a movable wheel with an adjustable tension mechanism. Both ends of the wire saw are attached to the wire drum pulley, and the wire direction is switched by two travel switches in the bottom of the wire drum pulley. The tension of the wire saw is adjusted by moving the movable wheel. The part is mounted on the workbench and fed towards the wire.

The proposed static normal force static model is [13]

(1) $$F_{n}\left(t\right)=K_{n}V_{x}{\left(t\right)}^{\alpha }V_{s}{\left(t\right)}^{\beta }$$

where *K_n_*, *α*, and *β* are experimentally determined coefficients. In this study, the cutting forces will be regulated by adjusting the part feed rate or wire saw velocity.

**Figure 3:**
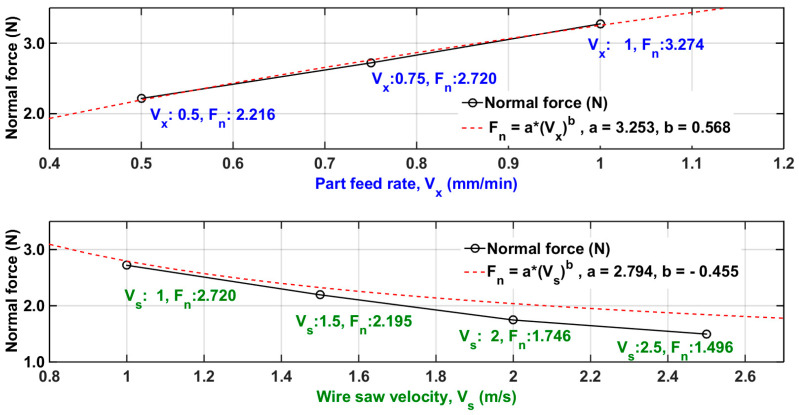
The experiment results of normal force with part feed rate and wire saw velocity.

In the following experiments, the wire saw velocity is 1.0, 1.5, and 2.0 m/s, the part feed rate is 0.5, 0.75, and 1.0 mm/min, the part length is 26 mm, and the wire saw tension force air source pressure is 0.20 MPa. The experiment results are shown in Figure 3.

The experiment is used to find the model coefficients *K*, *α*, and *β*. The model, developed by using least squares, is

(2) $$\begin{array}{l} F_{n}\left(t\right)=K\;V_{x}{\left(t\right)}^{\alpha },\;V_{s}=1.5,\;K=3.253,\;\alpha =0.568\\ F_{n}\left(t\right)=K\;V_{s}{\left(t\right)}^{\beta },\;V_{x}=0.75,\;K=2.794,\;\beta =-0.455 \end{array}$$

#### 2.2.2. Normal Force Controller Design

Linearizing Equation (1) yields

(3) $$\Delta F_{n}\left(t\right)=\left[K_{s}\beta {\left({\bar{V}}_{x,s}\right)}^{\beta -1}\right]\Delta V_{x,s}(t),$$

where the incremental normal force is $\Delta F_{n}\left(t\right)=F_{n}\left(t\right)-{\bar{F}}_{n}$, the nominal normal force is ${\bar{F}}_{n}={\bar{F}}_{r}$, ${\bar{F}}_{r}$ is the nominal reference force (N), the incremental velocity is $\Delta V_{x,s}\left(t\right)=V_{x,s}\left(t\right)-{\bar{V}}_{x,s}$, and the nominal velocity is

(4) $${\bar{V}}_{x,s}={\left[\frac{{\bar{F}}_{r}}{K_{s}}\right]}^{\left(1/\beta \right)},$$

The wire saw dynamics are expressed as Equation (5):

(5) $$\tau {\dot{V}}_{x,s}\left(t\right)=-V_{x,s}\left(t\right)+K_{w}V_{c}\left(t\right)$$

where *τ* is the time constant, which is determined experimentally, *K_w_* = 1 is the gain, and *V_c_* is the command velocity. Linearizing Equation (5) yields

(6) $$\tau \Delta {\dot{V}}_{x,s}\left(t\right)=-\Delta V_{x,s}\left(t\right)+K_{w}\Delta V_{c}\left(t\right)$$

where $\Delta V_{c}\left(t\right)=V_{c}\left(t\right)-{\bar{V}}_{c}$ is the incremental command velocity. The nominal command velocity is ${\bar{V}}_{c}={\bar{V}}_{x,s}$. The transfer function relating the incremental force to the incremental command velocity is

(7) $$\frac{\Delta F_{n}\left(s\right)}{\Delta V_{c}\left(s\right)}=\left[\beta {\left(\bar{V_{s,x}}\right)}^{\beta -1}\right]\frac{K_{s}}{\tau s+1}$$

The transfer function for a PI controller is

(8) $$\frac{\Delta V_{c}\left(s\right)}{\Delta E_{n}\left(s\right)}=\frac{K_{p}s+K_{i}}{s}$$

where *K_p_* is the proportional gain (m/s/N), Ki is the integral gain (m/s^2^/N), and the incremental force error is

(9) $$\Delta E_{n}\left(s\right)=\Delta F_{r}\left(s\right)-\Delta F_{n}\left(s\right)$$

By combining Equations (3)–(5), the closed-loop transfer function is obtained:

(10) $$\frac{\Delta F_{n}\left(s\right)}{\Delta F_{r}\left(s\right)}=\frac{\tau^{-1}\left(K_{p}s+K_{i}\right)K_{s}\beta {\left(\bar{V_{x,s}}\right)}^{\beta -1}}{s^{2}+\tau^{-1}\left[1+K_{p}K_{s}\beta {\left(\bar{V_{x,s}}\right)}^{\beta -1}\right]s+\tau^{-1}K_{i}K_{s}\beta {\left(\bar{V_{x,s}}\right)}^{\beta -1}}$$

The closed-loop system is designed to be overdamped with two time constants, *τ*_1_ and *τ*_2_, and the controller gains are

(11) $$\begin{array}{ccc} K_{p}=\frac{\left(\tau_{1}^{-1}+\tau_{2}^{-1}\right)\tau -1}{K_{s}\beta {\left(\bar{V_{x,s}}\right)}^{\beta -1}} & & K_{i}=\frac{\tau_{1}^{-1}\tau_{2}^{-1}\tau }{K_{s}\beta {\left(\bar{V_{x,s}}\right)}^{\beta -1}} \end{array}$$

**Figure 4:**
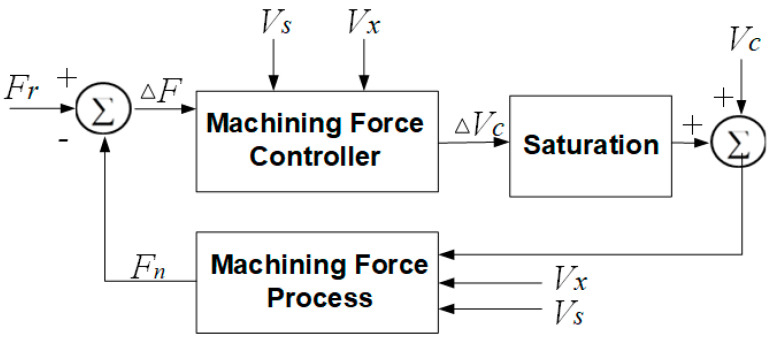
Normal force control system block diagram.

The controller difference equation is

(12) $$V_{c}\left(i\right)=V_{c}\left(i-1\right)+K_{p}\left[\Delta E_{n}\left(i\right)-\Delta E_{n}\left(i-1\right)\right]+K_{i}\Delta E_{n}\left(i\right)dt$$

where *dt* is the sample period in seconds. To ensure that integral windup does not occur, the incremental velocity is saturated via

(13) $$\begin{array}{ccc} \Delta V_{x,s}\left(t\right)=V_{\max}-{\bar{V}}_{x,s} & \mathrm{if} & \Delta V_{x,s}\left(t\right)\geq V_{\max}-{\bar{V}}_{x,s}\\ \Delta V_{x,s}\left(t\right)=V_{\min}-{\bar{V}}_{x,s} & \mathrm{if} & \Delta V_{x,s}\left(t\right)\leq V_{\min}-{\bar{V}}_{x,s} \end{array}$$

where *V_min_* = 0.5 m/s is the minimum wire saw velocity, and *V_max_* = 4 m/s is the maximum wire saw velocity. The implemented wire saw velocity is $V_{s}\left(t\right)=\Delta V_{s}\left(t\right)+{\bar{V}}_{s}$. The control system block diagram is given in Figure 4.

### 2.3. Controller Parameter Determination

Through the establishment of the above closed-loop transfer function model, the control parameters *K_p_* and *K_i_* of the PI controller are related to the time constant *τ* of the transfer function of the open-loop system and the time constants *τ*_1_ and *τ*_2_ of the closed-loop transfer function. The correlation coefficients need to be determined by experiments and simulations.

In the diamond wire saw cutting system, the workpiece feed and wire saw velocity execution units were a stepping motor and a DC motor, respectively, which can be simplified as a first-order inertial system. In order to obtain the time constant of the system’s time-domain performance index, the system is analyzed in the time domain. A specific input signal is applied to the system, and the system performance index is obtained by measuring the time response of the system. The step response is selected here for measurement.

Step functions are defined as:

(14) $$r(t)=\left\{ \begin{array}{l} 0,t<0\\ A,t\geq 0 \end{array}\right.$$

where *r*(*t*) represents the output value of this step function *t*, and A represents the step value of this step function.

The step function is a segmented function. In the actual measurement, the power supply is turned on, and the mutation of the motor load has the characteristics of a step function. Here, the motor power-on is applied to maintain a constant command control signal at rest, and the command control signal may be considered as an input step signal. Thus, Equation (15) may represent the command velocity. The Laplace transform of the step signal is

(15) $$R(s)=\frac{A}{s}$$

The system being tested can be represented by a first-order inertial system with a transfer function model:

(16) $$G(s)=\frac{Y(s)}{R(s)}=\frac{1}{\tau s+1}$$

The above two forms are merged, and the inverse Laplace transform is performed, yielding the following:

(17) $$y(t)=A(1-e^{-\frac{t}{\tau }})$$

**Figure 5:**
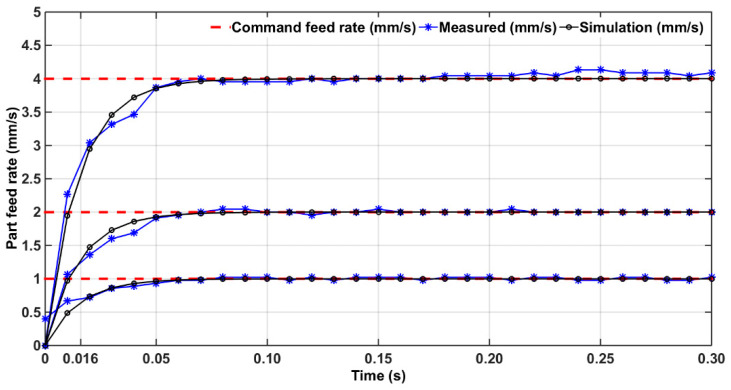
Feed rate measurement for determining time constant.

Therefore, the corresponding abscissa value of the measured step response curve is the system time constant *τ*. Here, the sampling frequency was 100 Hz, and the command velocity was selected as 1, 2, or 4 mm/s. The experimental results are shown in Figure 5, and the time constant was about 0.016 s.

**Figure 6:**
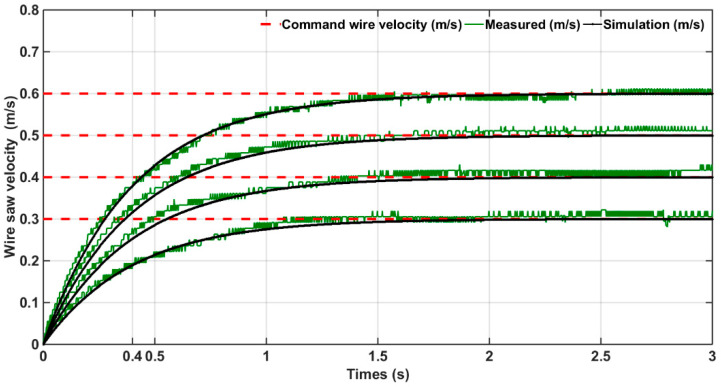
Wire velocity measurement for determining time constant.

Similarly, the wire saw speed was selected as 0.3, 0.4, 0.5, and 0.6 mm/s, The experimental results are shown in Figure 6, and the time constant of the measured wire saw speed was about 0.4 s.

## 3. Experimental Studies

According to the established control model, the control program written in LabView with the PXIe-6358 multifunction acquisition card as its core completed the implementation of the control experiment based on the previously mentioned experimental platform. This program integrated the force sensors and the control system of multiple execution units. Using the PI control model in the control software, the control program computed the corresponding command velocity after obtaining the cutting force from the force sensor. In addition, it is inevitable that the wire saw will exhibit wear in the process of wire saw cutting, and the experiment in this paper is mainly to compare and analyze the control effect of different control objects, and use a new wire saw as much as possible in the actual experiment to reduce the impact of wire saw wear on the experimental effect.

### 3.1. Normal Force Control Experiment with Feed Rate

#### 3.1.1. Control Experiment Result with Feed Rate

**Figure 7:**
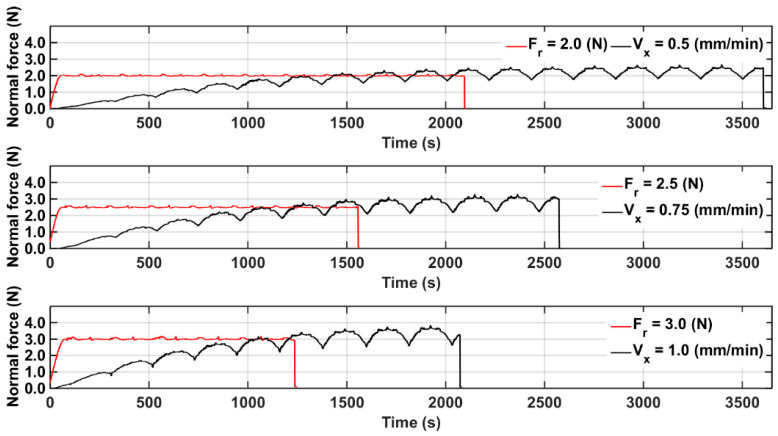
Normal force profile of single-crystal silicon with constant cutting parameters (*V_s_* = 1 m/s) and part feed rate control.

In each experiment, the part size and process parameters were the same as those used in the previous experiments. In order to investigate the controller performance during the entire silicon wafer cutting process for different normal forces, experiments utilizing reference normal forces of 2.0, 2.5, and 3.0 N were conducted, with corresponding part feed rates of 0.5, 0.75, and 1.0 mm/min, and the wire saw velocity was 1 m/s. The controller gains *K_p_* and *K_i_* were −5 and −0.001, respectively. The comparison of the forces is shown in Figure 7.

**Figure 8:**
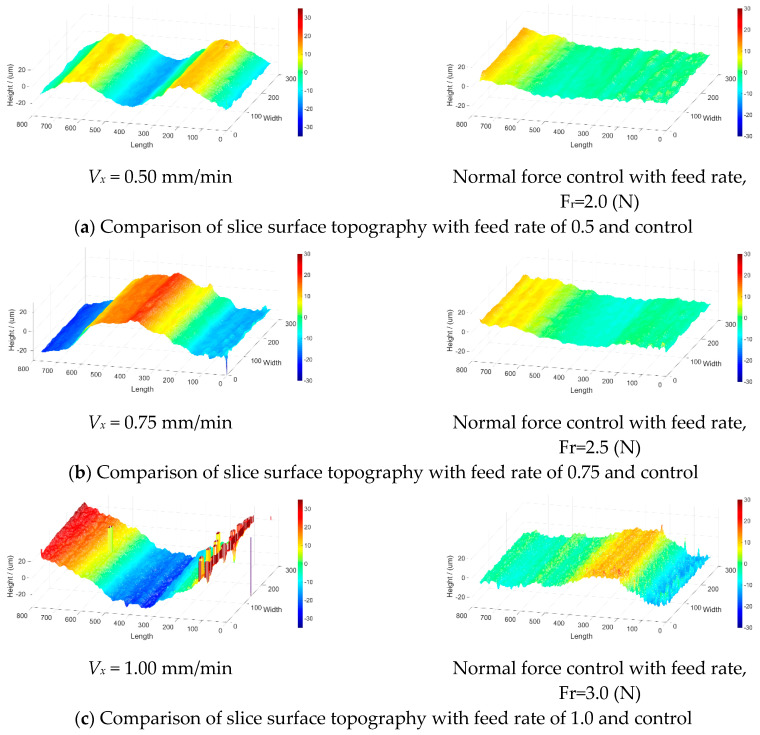
Surface roughness of single-crystal silicon with feed rate control.

The average surface roughness values were 7.87, 12.42, and 16.25 μm for constant part feed rates of 0.50, 0.75, and 1.00 mm/min, respectively. The values were 3.16, 4.32, and 5.70 μm when controlling the reference normal force at values of 2.0, 2.5, and 3.0 N, respectively. Figure 8a–c show the part surface topologies for part feed rates of 0.50, 0.75, and 1.00 mm/min, respectively, and the corresponding surface topologies when using normal force control. The wire velocity of these experiments is 1.0 m/s.

The variations in the surface profiles dramatically increased as the part feed rate increased, which was because the processing forces increased. Furthermore, variations in the surface profiles dramatically decreased with the use of force control because the force control substantially decreased the force variations. The part average surface roughness values with and without control are shown in Table 2, as are the total operation times. The data showed that force control was able to reduce surface roughness by 60% compared to that with the constant part feed rate, and the total operation time was decreased by about 40%.

#### 3.1.2. Control Analysis of Feed Rate

Based on the comparison of the difference between the measured and reference cutting forces, the computer sent different pulse counts to the step motor drive at each sample period and adjusted the part feed rate accordingly.

**Figure 9:**
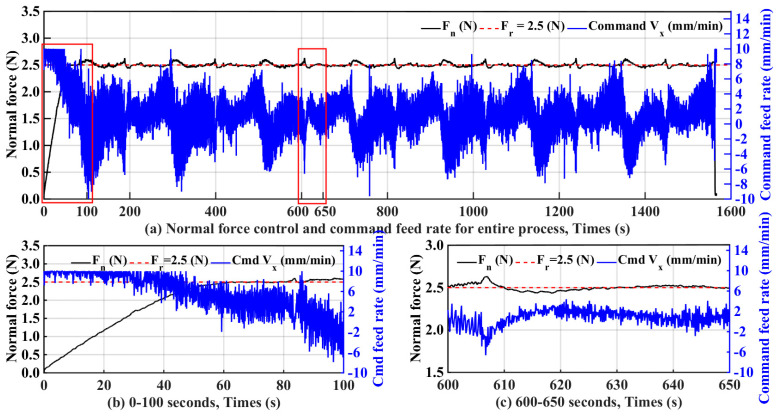
Normal force control and command feed rate for entire process with F_r_ = 2.5 N.

In the initial stage of cutting, as shown in Figure 9b, the workpiece and the wire saw had not yet fully contacted, and the normal cutting force gradually began to become larger than 0 but was far from reaching the reference force value. At this time, the feed rate had reached the maximum. Here, the maximum value of the feed rate was 10 (mm/min). It is known from the figure that the cutting force was close to the reference force of 2.5 (N) until the feed speed was maintained close to the maximum feed rate.

In the cutting process, the tension of the wire saw undergoes a process of change, so the cutting force caused by the workpiece will also change. The results for cutting times of 600–650 s are shown in subplot at the bottom right of Figure 9c. The measured normal force value at 610 s varied significantly. The force value was higher than the reference force. At this time, the feed speed of the workpiece had a significant decrease, making the normal force smaller. When the normal force was less than the reference force after 610 s, the feed rate gradually became larger, causing the cutting force to grow slowly to close to the reference value of the normal cutting force. In this way, the value of the cutting force was adjusted until the end of the entire cutting process.

**Figure 10:**
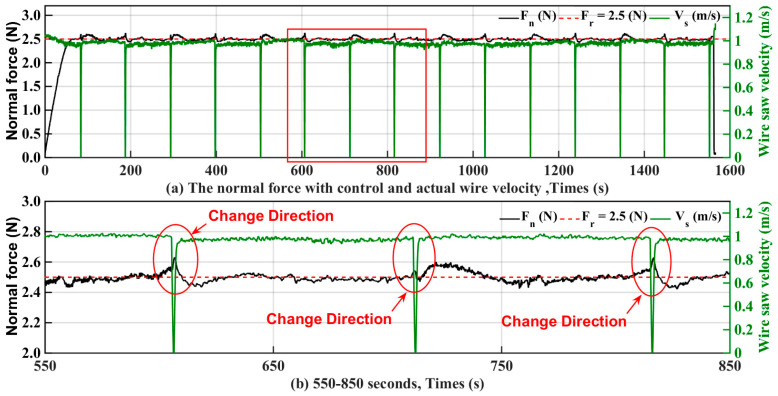
Relationship between normal force with control and actual wire velocity.

Figure 10a shows the relationship between the normal force with control and the actual wire velocity. It is clear that at each wire saw cycle, as shown in Figure 10b (that is, when the wire saw velocity decreased to 0 and then became larger), there was a significant variation in the normal cutting force, which was caused by the design structure of the wire saw cutting machine itself.

### 3.2. Normal Force Control Experiment of Wire Velocity

#### 3.2.1. Control Experiment Result via Wire Saw Velocity

Experiments utilizing reference normal forces of 2.3, 2.0, and 1.7 N were conducted, which corresponded to the normal forces for wire velocities of 1, 1.5, and 2 m/s, respectively, and the part feed rate was 0.75 mm/min. The controller gains *K_p_* and *K_i_* were −5 and −0.001, respectively.

**Figure 11:**
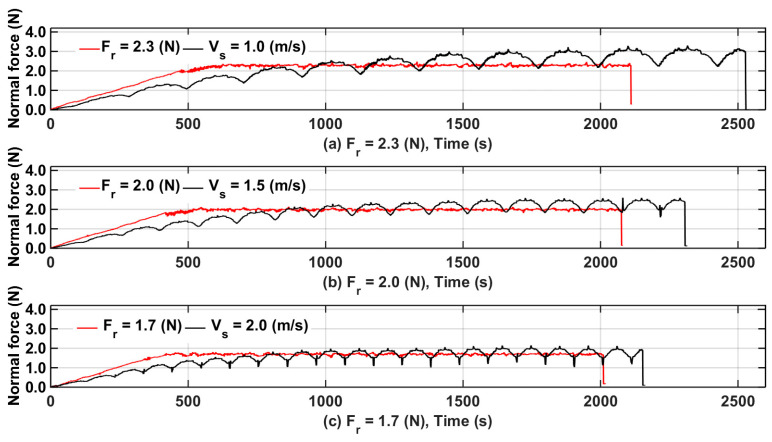
Normal force profile of silicon with constant cutting parameters (Vx = 0.75 mm/min) and wire saw velocity control.

Figure 11a–c show the control results when the reference normal force was 2.3, 2.0, and 1.7 N. The controller began to regulate the wire velocity at 800, 650, and 470 s when the reference normal forces were 2.3, 2.0, and 1.7 N, respectively. The force fluctuations occurred because the wire saw changed direction constantly. When each wire saw changed direction, the force fluctuated up and down, which caused the difference between the measured force and reference force to change constantly, indicating that the controller worked well.

**Figure 12:**
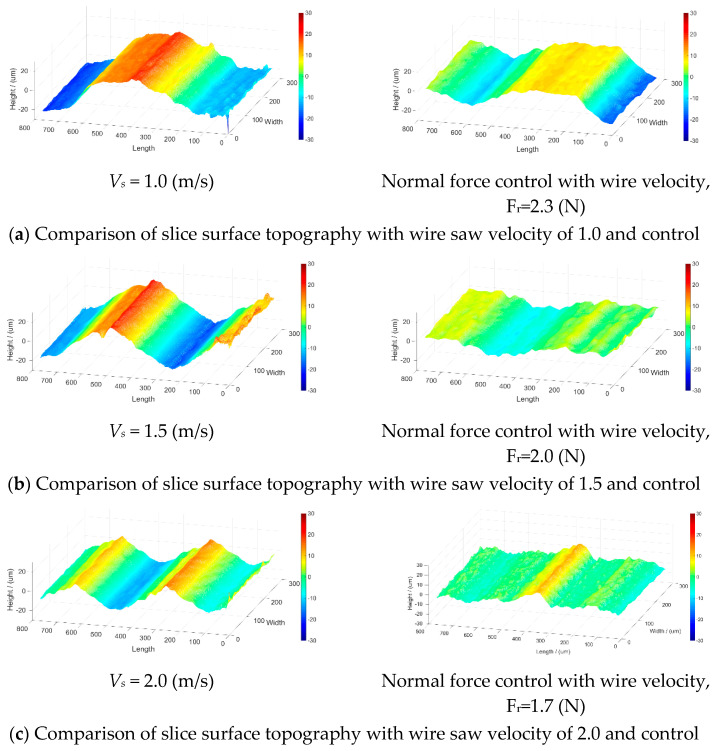
Surface roughness of mono-crystal silicon with wire velocity control.

Based on the surface roughness plots in Figure 12a–c, the surface roughness values with the controlled wire velocity were smaller. The part feed rate is 0.75 mm/min.

Figure 12a–c show the comparison of the surface roughness values of optical glass with a feed rate of 0.75 mm/min and wire velocities of 1, 1.5, and 2 m/s. The surface roughness values were 12.42, 9.98, and 6.11 µm for a constant part feed rate of 0.75 mm/min and wire velocities of 1, 1.5, and 2 m/s, respectively. On the other hand, when using the PI controller with a part feed rate of 0.75 mm/min and reference cutting forces of 2.5, 2.0, and 1.7 N, the surface roughness values were 6.85, 3.53, and 2.85 μm, respectively. The cutting times and surface roughness values with and without wire velocity control are compared in Table 3. As shown in Table 3, the cutting time differences between the cases with constant and varying wire velocities were very small, ranging from 7.2% to 17.6%. The reason was that the constant feed rate led to the same cutting time. The surface roughness between the cases with a constant and varying wire velocity changed significantly, with approximately a 50% increase on average. The reason was that the controlled wire velocity made the cutting process more stable and the cutting force fluctuations smaller than the constant wire velocity, which led to smaller surface roughness values.

#### 3.2.2. Control Analysis of Wire Saw Velocity

**Figure 13:**
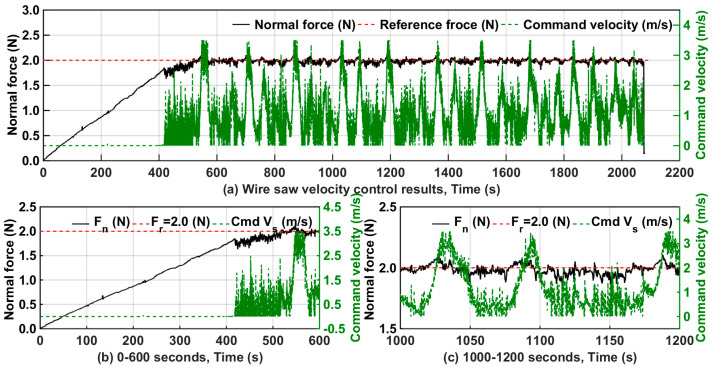
Wire saw velocity control results when reference normal force was 2.0 N.

For F_r_ = 2.0 N, which corresponds to a constant wire velocity of 1.5 m/s, the plot of the normal force and command wire velocity is given in Figure 13. As Figure 13b shows, notice that the commanded wire velocity substantially increases when the wire switches direction and that a constant normal force is maintained even during steady cutting when the commanded wire velocity experiences saturation. Also, the wire velocity saturates at its lower level when the wire first engages the part since the normal force is low and continues until the length of contact between the wire and part nearly reaches a steady state.

In the process time of 1000–1200 s represented in the lower right of Figure 13c, the 1050 s front force value underwent a significant increase, and the measured normal force value was higher than the reference force. At this time, the wire saw velocity significantly increased to the maximum set velocity, and the cutting force became smaller. After 1050 s, the cutting force was less than the reference force, and the wire saw speed gradually decreased. The cutting force grew slowly to a value near the reference value of the normal cutting force. The magnitude of the normal cutting force was varied in this way until the end of the entire cutting process.

### 3.3. Comparison and Analysis

**Figure 14:**
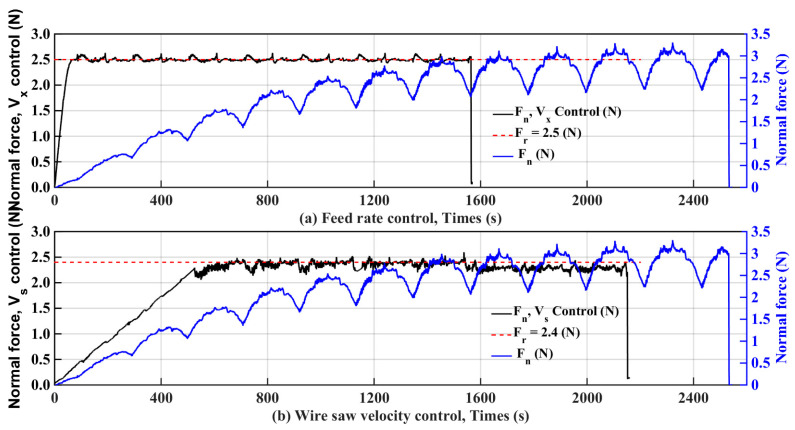
Feed rate versus wire saw velocity control.

Through the control of the feed rate and the adjustment of the wire saw velocity, the cutting process can be completed with a given reference force, and the cutting efficiency and cutting quality were improved compared to those in the case with fixed cutting parameters. The different cutting forces are shown in Figure 14a,b.

Table 4 presents the processing time after increasing the wire saw speed controller in the three sets of experiments with controlled saw speeds. The increase was 17.6%, 10.2%, and 7.2%, respectively, with an average value of 11.7%. Moreover, the surface roughness increased by 44.8%, 64.6%, and 53.3%, respectively, with a mean of 54.2%.

In the three sets of experiments with feed rate control, the processing time after increasing the feed rate controller was increased by 42.0%, 39.6%, and 40.1%, and the average value was 40.6%. The surface roughness value decreased by 59.8%, 65.2%, and 64.9%, with an average of 63.3%. Therefore, the control of the feed speed was better than the control of the saw speed.

## 4. Summary and Conclusions

In this paper, the effects of the wire saw speed and workpiece feed rate on the cutting force in the diamond wire saw cutting of monocrystalline silicon were studied. The cutting force was varied in the experimental studies using a PI controller. Tests were conducted for square monocrystalline silicon processing. The results showed that the PI controller could be effectively applied to maintain a constant force during the cutting process. Under a constant applied cutting force, the control of the feed rate and the wire saw cutting speed would affect cutting time and surface finish of the cut specimen.

The results showed that the wire saw processing with force control significantly reduced the surface roughness of the wafer, compared to constant feed and saw speeds. Average reductions of about 63.3% under feed speed control and 54.2% under wire saw speed control were achieved. However, the PI controller with feed speed control had an improved cutting efficiency, averaging at about 40.6%.

The control of feed speed and wire saw speed can also increase a certain cutting efficiency, according to the results of the two control experiments. However, the cutting force jump point in the cutting process still exists, so future considerations regarding how to combine the two controlled objects can help achieve the goal of removing the cutting force jump point in the cutting process.

## Figures and Tables

**Table 1:** Technical parameters of sand wire cutting machine.

Parameter	Value	Parameter	Value
Workbench stroke (mm)	120 × 120	Feed rate (mm)	0–3 mm/min
Wire saw diameter (mm)	0.24	Wire saw velocity (m/s)	0–4
Abrasive size (μm)	30–50	Tension (MPa)	0–0.6

**Table 2:** Comparison of feed speed control and fixed-parameter experiments.

Constant Feed Rate and Wire Saw Velocity				Controlled Wire Velocity			Comparison (%)	
Feed Rate (mm/min)	Normal Force (N)	Time (s)	Surface Roughness (μm)	Reference Force (N)	Time (s)	Surface Roughness (μm)	Time (s)	Surface Roughness (μm)
0.5	2.216	3618	7.87	2.0	2098	3.16	42.0	59.8
0.75	2.720	2538	12.42	2.5	1561	4.32	39.6	65.2
1.0	3.274	2081	16.25	3.0	1246	5.70	40.1	64.9

**Table 3:** Comparison of wire saw velocity control and fixed-parameter experiments.

Constant Wire Velocity				Control Wire Velocity			Comparison (%)	
Wire Velocity (m/s)	Normal Force (N)	Time (s)	Surface Roughness (μm)	Reference Force (N)	Time (s)	Surface Roughness (μm)	Time (%)	Surface Roughness (%)
1	2.720	2538	12.42	2.5	2091	6.85	17.6	44.8
1.5	2.195	2314	9.98	2.0	2079	3.53	10.2	64.6
2	1.746	2161	6.11	1.7	2006	2.85	7.2	53.3

**Table 4:** Comparison of feed speed and wire saw velocity control.

Name	Feed Rate Control Experiment Efficiency Improvement (%)				Wire Saw Velocity Control Experiment Efficiency Improvement (%)			
Number	1	2	3	Average	1	2	3	Average
Time	42.0	39.6	40.1	40.6	17.6	10.2	7.2	11.7
Sa	59.8	65.2	64.9	63.3	44.8	64.6	53.3	54.2

## Data Availability

All data generated or analyzed during this study are included in this published article.

## Nomenclature

*dt*	Sample period (s)
*F_n,t_*	Normal or tangential force error (N)
${\bar{F}}_{n,t}$	Nominal Normal or tangential force (N)
*F_r_*	Reference force (N)
$\bar{F_{r}}$	Nominal reference force (N)
*F_n_*	Normal force acting on wire saw (N)
*K_n_, K, K_p_*	Coefficient
*K_w_*	Gain of commanded wire saw velocity
P	Tension force (N)
*V_c_*	The command wire velocity (m/s)
$\bar{V_{c}}$	Nominal command wire velocity (m/s)
*V_max_*	Maximum wire saw velocity (m/s) or part feed rate (mm/min)
*V_min_*	Minimum wire saw velocity (m/s) or part feed rate (mm/min)
*V_s_*	Wire saw velocity (m/s)
${\dot{V}}_{x,s}$	Wire saw (m/s) of part feed rate (mm/min) dynamic variation
$\bar{V_{x,s}}$	Nominal wire velocity (m/s) or part feed rate (mm/min)
*V_x_*	Part feed rate (mm/min)
α, β	Coefficient
$\Delta E_{n}$	Incremental normal force error (N)
$\Delta F_{n}$	Incremental normal force (N)
$\Delta F_{r}$	Incremental reference force (N)
$\Delta V_{x,s}$	Incremental part feed rate (mm/min) or wire velocity (m/s)
$\Delta V_{c}$	Incremental command wire velocity
τ	Time constant (s)
$\tau_{1},\;\tau_{1}$	Time constants of closed-loop overdamped system (s)
